# Occupational accidents among the nurses

**DOI:** 10.1186/1753-6561-5-S6-P288

**Published:** 2011-06-29

**Authors:** A Özarslan

**Affiliations:** 1 Infection Control Commitee, Gülhane Military Medical Academy, Ankara, Turkey

## Introduction / objectives

This is a cross sectional study designed to define factors which affect the frequency, distribution, types and notification of occupational accidents in the nurses working in Gulhane Military Medical Academy.

## Methods

In this study, 504 of 570 nurses were reached. All of these nurses were applied a questionnaire related with occupational accidents. An observational study was made for occupational accidents, which they experienced 2009, were performed. The accidents were detected either by using a notification form or by periodical observations during the period. Arithmetic mean and frequency analyses were used for analyzing the data and chi-square test was applied for comparison of the groups. In the analysis a=0,005 value was chosen as error margins.

## Results

According to results of study; 43.1 % of the nurses declared they experienced an occupational accident in the last year, 53 % declared individual protective equipment was partially supplied. The 50 % of the participants stated only a moderate level of safety precautions was provided due to their occupational risks. In a three-month period, frequency rate of occupational accidents was 8.1 %, and density rate was 24/1000 per 100 hours. Sharp injury accident was the first with 70.7 %. The most frequent exposure type was the needle-stick injury of hand fingers.Regarding the accident as unimportant was the main reason for ignoring declaration. No attempt for medical advice was made for thirty (73.1 %) accidents. The risk for occupational accidents was increased with the overtime working (p=0.039). The nurses experiencing occupational accidents realize the preventive measures for occupational risks to be at moderate level (p=0.011).

## Conclusion

In conclusion, the educational level of the nurses for occupational health and safety are not adequate. Occupational health and safety should be under the supervision of a board, which must include must include experts.

## Disclosure of interest

A. Özarslan Other MSN

